# Expression of genes regulating cell division in porcine follicular granulosa cells

**DOI:** 10.1186/s13008-023-00094-7

**Published:** 2023-08-07

**Authors:** Jakub Kulus, Wiesława Kranc, Magdalena Kulus, Piotr Dzięgiel, Dorota Bukowska, Paul Mozdziak, Bartosz Kempisty, Paweł Antosik

**Affiliations:** 1 Department of Diagnostics and Clinical Sciences, Institute of Veterinary Medicine, Nicolaus Copernicus University in Torun, Torun, Poland; 2 Department of Anatomy, Poznan University of Medical Sciences, Poznan, Poland; 3 Department of Veterinary Surgery, Institute of Veterinary Medicine, Nicolaus Copernicus University in Torun, Torun, Poland; 4 Division of Histology and Embryology, Department of Human Morphology and Embryology, Wroclaw Medical University, Wroclaw, Poland; 5 Department of Physiotherapy, Wroclaw University School of Physical Education, Wroclaw, Poland; 6 Physiology Graduate Faculty, College of Agriculture and Life Sciences, North Carolina State University, Raleigh, NC 27695 USA; 7 Division of Anatomy, Department of Human Morphology and Embryology, Wroclaw Medical University, Wroclaw, Poland; 8 Center of Assisted Reproduction, Department of Obstetrics and Gynecology, University Hospital and Masaryk University, Brno, Czech Republic

**Keywords:** Follicular granulosa cells, Cellular signaling, Cytoskeleton organization, Cell cycle, Gene expression profile, Transcriptomics

## Abstract

**Background:**

Cell cycle regulation influences the proliferation of granulosa cells and affects many processes related to ovarian folliclular growth and ovulation. Abnormal regulation of the cell cycle can lead to many diseases within the ovary. The aim of this study was to describe the expression profile of genes within granulosa cells, which are related to the formation of the cytoskeleton, organization of cell organelles inside the cell, and regulation of cell division. Established in vitro primary cultures from porcine ovarian follicle granulosa cells were maintained for 48, 96, 144 h and evaluated via microarray expression analysis.

**Results:**

Analyzed genes were assigned to 12 gene ontology groups "actin cytoskeleton organization", "actin filament organization", "actin filament—based process", "cell—matrix adhesion", "cell—substrate adhesion", "chromosome segregation", "chromosome separation", "cytoskeleton organization", "DNA integrity checkpoint", "DNA replication initiation", "organelle fision", "organelle organization". Among the genes with significantly changed expression, those whose role in processes within the ovary are selected for consideration. Genes with increased expression include (ITGA11, CNN1, CCl2, TPM2, ACTN1, VCAM-1, COL3A1, GSN, FRMD6, PLK2). Genes with reduced expression inlcude (KIF14, TACC3, ESPL1, CDC45, TTK, CDC20, CDK1, FBXO5, NEK2—NIMA, CCNE2). For the results obtained by microarray expressions, quantitative validation by RT-qPCR was performed.

**Conclusions:**

The results indicated expression profile of genes, which can be considered as new molecular markers of cellular processes involved in signaling, cell structure organization. The expression profile of selected genes brings new insight into regulation of physiological processes in porcine follicular granulosa cells during primary in vitro culture.

## Background

The ability of animal cells to divide, transmit signals, move, and perform metabolic activity depends on the cytoskeleton. In addition, the cytoskeleton is responsible for cell shape, durability, and resistance to compression [1]. Depending on the environmental conditions, the cell, has the ability to adjust its shape, whether it is in vitro or in vivo [2]. The cytoskeleton is a dynamic structure, modifying its composition continuously. Components that build the cytoskeleton include microfilaments, microtubules, and intermediate filaments. The structural protein that builds microfilaments is actin, which is the most common protein in cells. The functions performed by microfilaments are numerous and include cell movement, intracellular signaling, and cell division [3], but also has been shown to be significantly involved in endocytosis [4]. Microtubules are made of the protein tubulin, are responsible for the transport of substances [5], and form the karyokinetic spindle responsible for the spread of chromosomes to the daughter cells [6]. Polyglutamination of tubulin also affecting the shape of the nucleus, according to recent data [7]. Intermediate filaments made of proteins such as vimentin, keratin and lamin are responsible for the majority of cell shape determination and cell stability but also for cell signaling [8]. In addition to functions related to stabilization of the cell environment, intermediate filaments also show activity in apoptosis, migration, adhesion, and interactions with other cytoskeletal components [2]. A functionally active animal cell requires the correct interactions of cytoskeletal components. Changes in the ratio of individual components of the cytoskeleton are described in diseases including cancer [9].

Granulosa cells (GCs) are the largest population of cells that make up the ovarian follicle. They have been shown to be intimately involved in the processes of folliculogenesis and oogenesis [10, 11]. Granulosa cells are responsible for steroidogenesis and their dialogue with an oocyte leads to its competence for fertilization [12]. The cytoskeleton involved in the cell division [13] influences the proliferation of granulosa cells in the ovarian follicle. The cytoskeleton has been shown to influence steroidogenesis in rat granulosa cells [14] through cholesterol transport [15, 16] and influence on localization of cell organelles [5, 17], including mitochondria in the cytoplasm [18]. A very important role of the cytoskeleton has been demonstrated in meiotic division of the oocyte [19, 20]. This results in an asymmetric division and the formation of a haploid oocyte and two polar bodies allowing the oocyte to retain the maternal components necessary for the initial development of the embryo. Critical steps in the above division are positioning of the nucleus, formation and migration of spindles, segregation of chromosomes, and extrusion of polar bodies, in which actin filaments are involved [19]. The processes involved in cell division, which is part of the cell cycle, are important for cell proliferation. The transition between the different phases G1, S, G2, M in the cell cycle is regulated by many genes and signaling pathways [21]. Cell division includes both cytoplasm and cell nucleus division, which must be controlled by checkpoints [22]. Abnormalities occurring during cell division can result in the formation of defective cells and consequently cell death. A number of genes responsible for cell division have been shown to be expressed, which if not properly expressed can cause the processes of tumorigenesis [23].

Additionally, intracellular and extracellular signaling is very important in processes related to animal reproduction and its efficiency [24]. The cytoskeleton and cell adhesion molecules (integrins, cadherins), catenins but also extracellular matrix (ECM) are involved in cell signaling [25, 26]. The orientation of the division spindle depends on extracellular matrix proteins confirming the interplay between the ECM and the cytoskeleton in cell division [27]. The main transmembrane proteins involved in the cell division are the integrins, which bind to cytoskeletal actin filaments [28]. A major role for these transmembrane proteins in reproduction in animals has been demonstrated [29]. Additionally, the recently described ability of granulosa cells to differentiate into other cell lineages gives these cells additional value [30, 31]. Small Rho GTPases affect cytoskeleton composition while showing effects on mesenchymal cell differentiation into adipose or muscle cells [32, 33]. Also the recently described effect of actin remodeling on the ability of mesenchymal stem cells to differentiate requires further research [34].

The aim of this study was to evaluate the expression profile of genes related to the formation of cytoskeleton, which is involved in cell division, spatial organization of cell organelles, metabolic activity and intercellular signaling in porcine granulosa cells. Describing the expression of individual genes related to intercellular signaling in granulosa cells may contribute to understanding the molecular basis of these cellular processes. Given the ability to co-culture granulosa cells along with oocytes, these data may be used in future studies to improve the efficiency of in vitro assisted reproductive techniques.

## Results

**Fig. 1:**
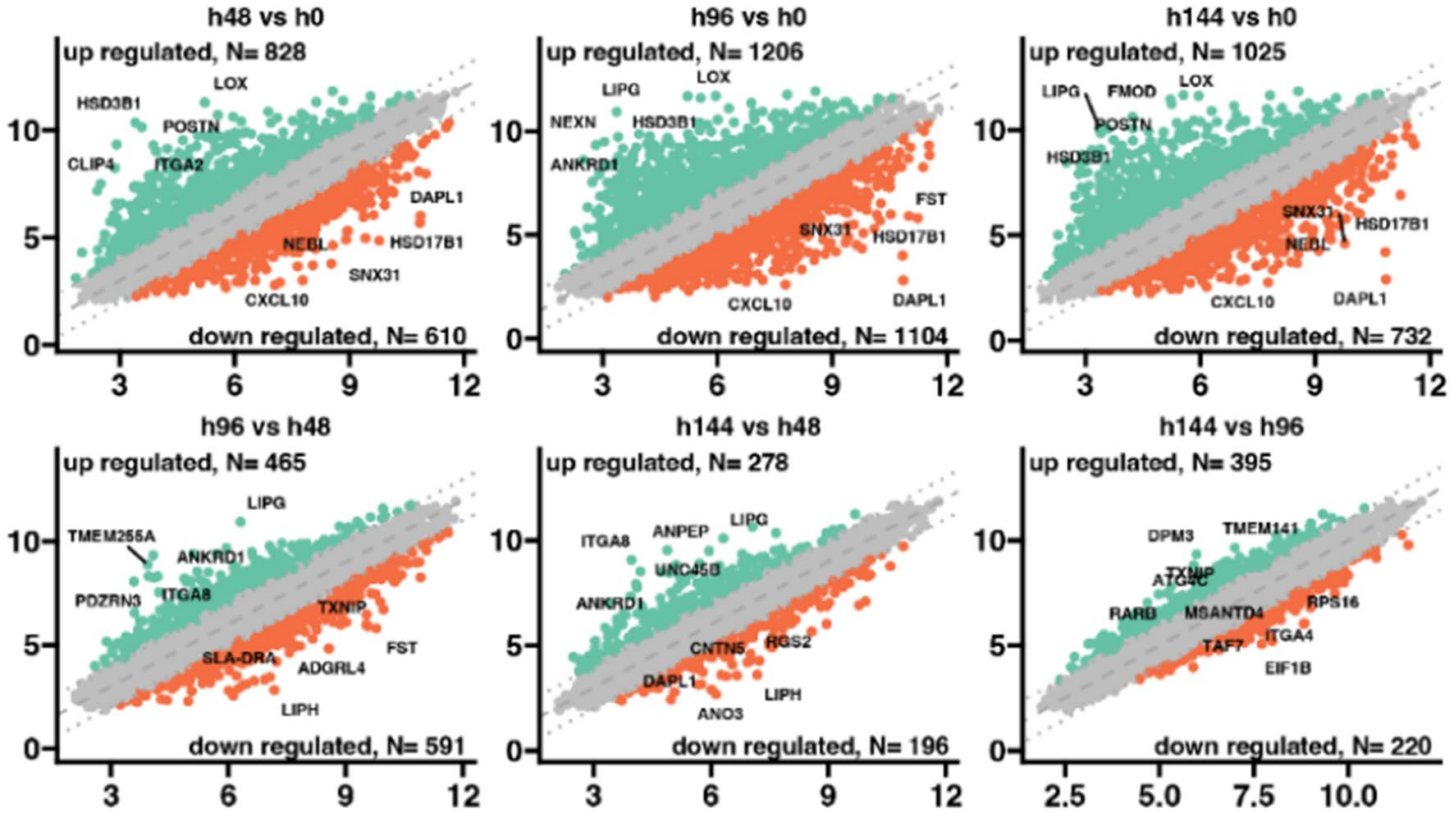
Distribution of differentially expressed genes visualized as scattered plots. Each dot represents the mean expression (two biological replicates) of an individual gene obtained from a normalized microarray study. The gray dotted lined (cut-off values) were established according to the following parameters: |fold change|= 2 and p value = 0.05. Genes above the cut-off lines were considered as differentially expressed genes and are shown as orange (down-regulated) and turquoise (up-regulated) dots. The total numbers of up- and down-regulated genes are given in the top right and top left corners, respectively. The symbols of the five most differentially expressed genes from each compression are marked on the plots

The porcine granulosa cells were collected at 48, 96, and 144-h of cultivation and compared to the 0-h of the experiment as a control group. The general profile of the transcriptome changes is shown in Fig. 1, where dots represent the mean gene expression. Concerning the cut-off criteria for differentially expressed genes (|fold change|> 2, and p value < 0.05), the scattered plots indicate that on 48-h of the experiment 828 genes were upregulated, while 610 were downregulated in comparison to 0 h of cells cultivation. At 96-h, it was revealed that 1206 activated and 1104 activated genes were, while at the end of the experiment at 144-h, it was indicated that 1025 upregulated and 732 downregulated genes in comparison to 0-h of the experiment. It was found that the most changes have been indicated during the 96-h of the experiment compared to the control. Meanwhile, the lowest number of deregulated genes were revealed between 48-h and controls. Commonly expressed genes were also identified across all analyzed groups. The expression of LOX, and HS3B1 were upregulated, and the expression of HSD17B1, SNX31, DAPL1, and CXCL10 were inhibited in all experimental groups compared to the control. Expression changes were also analyzed between experimental groups. It has been indicated that at 96- and 144-h groups ANKRD1, and ITGA8 genes are upregulated compared to 48-h of the experiment.

**Fig. 2:**
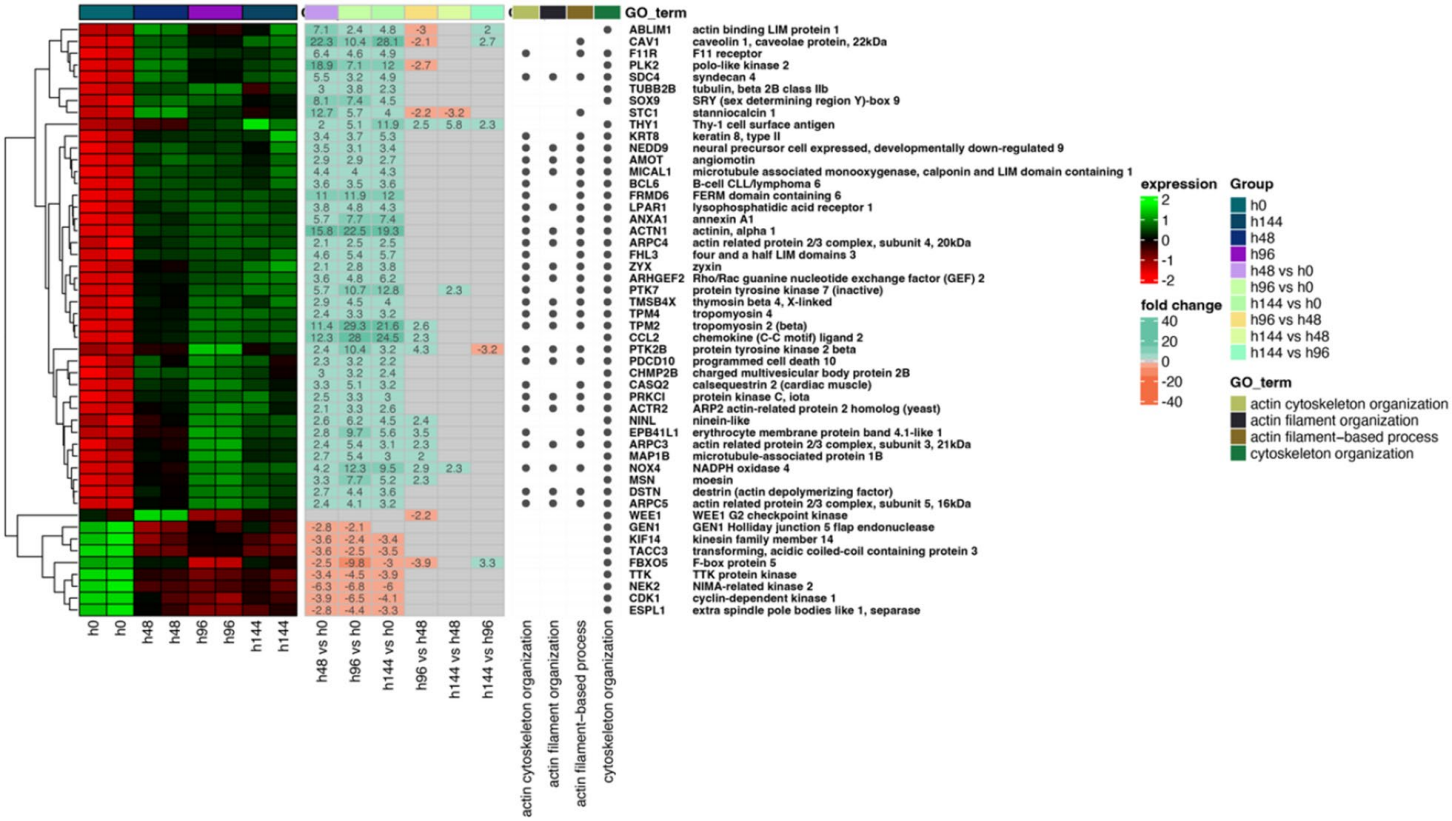
Heatmap of expressed genes related to actin filament and cytoskeleton organization. The heatmap shows the expressed genes (left side), expression fold changes (center), and GO term ontological groups with gene names (right side) of all analyzed groups. The legends on the right side illustrate the colors used for the visualization

**Fig. 3:**
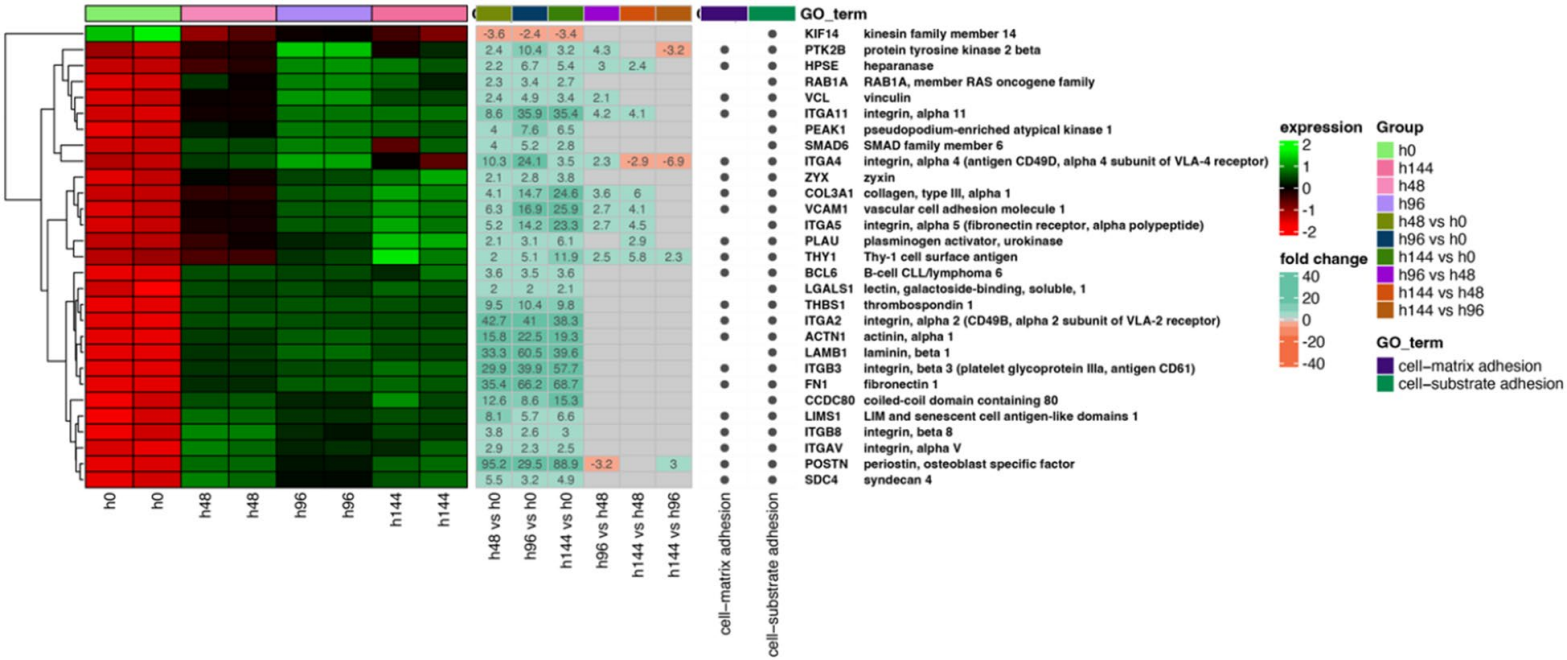
Heatmap of expressed genes related to cell–matrix and cell-substrate adhesion, according to the GO term. The heatmap shows the expressed genes (left side), expression fold changes (center), and GO term ontological groups with gene names (right side) of all analyzed groups. The legends on the right side illustrate the colors used for the visualization

**Fig. 4:**
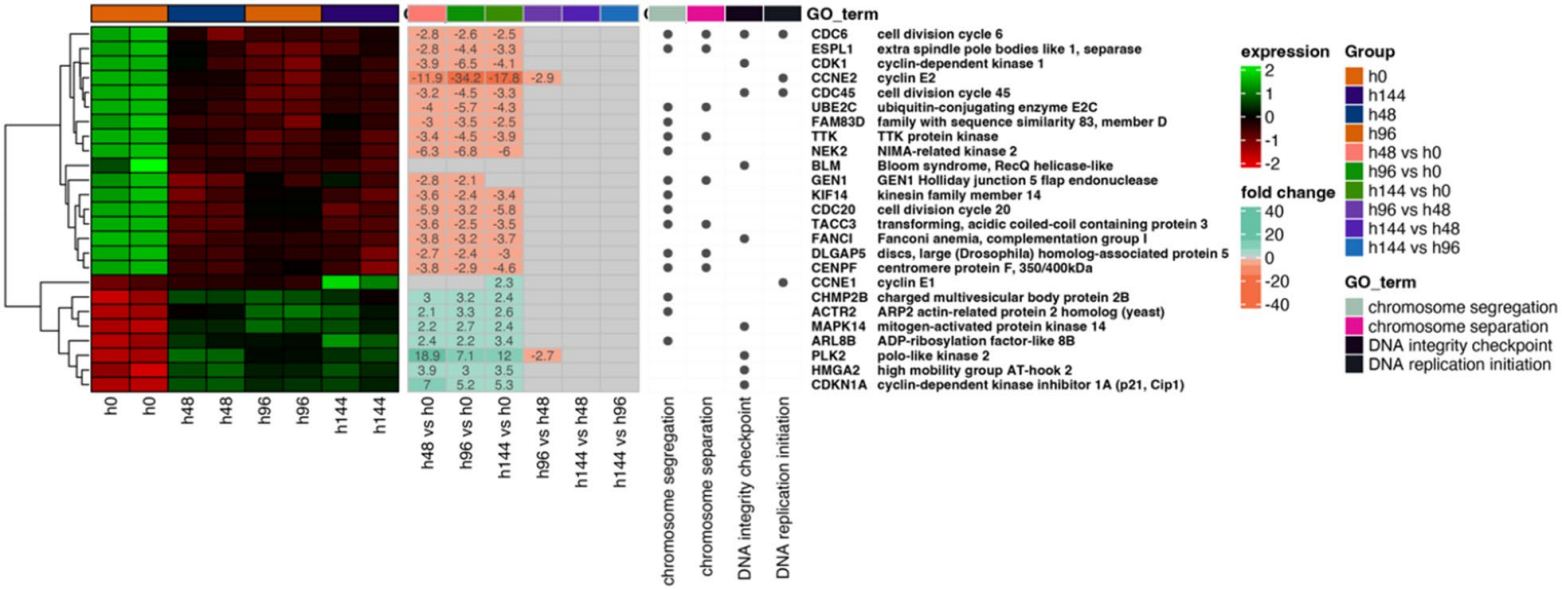
Heatmap of expressed genes related to chromosome segregation and DNA replication, according to the GO term. The heatmap shows the expressed genes (left side), expression fold changes (center), and GO term ontological groups with gene names (right side) of all analyzed groups. The legends on the right side illustrate the colors used for the visualization

**Fig. 5:**
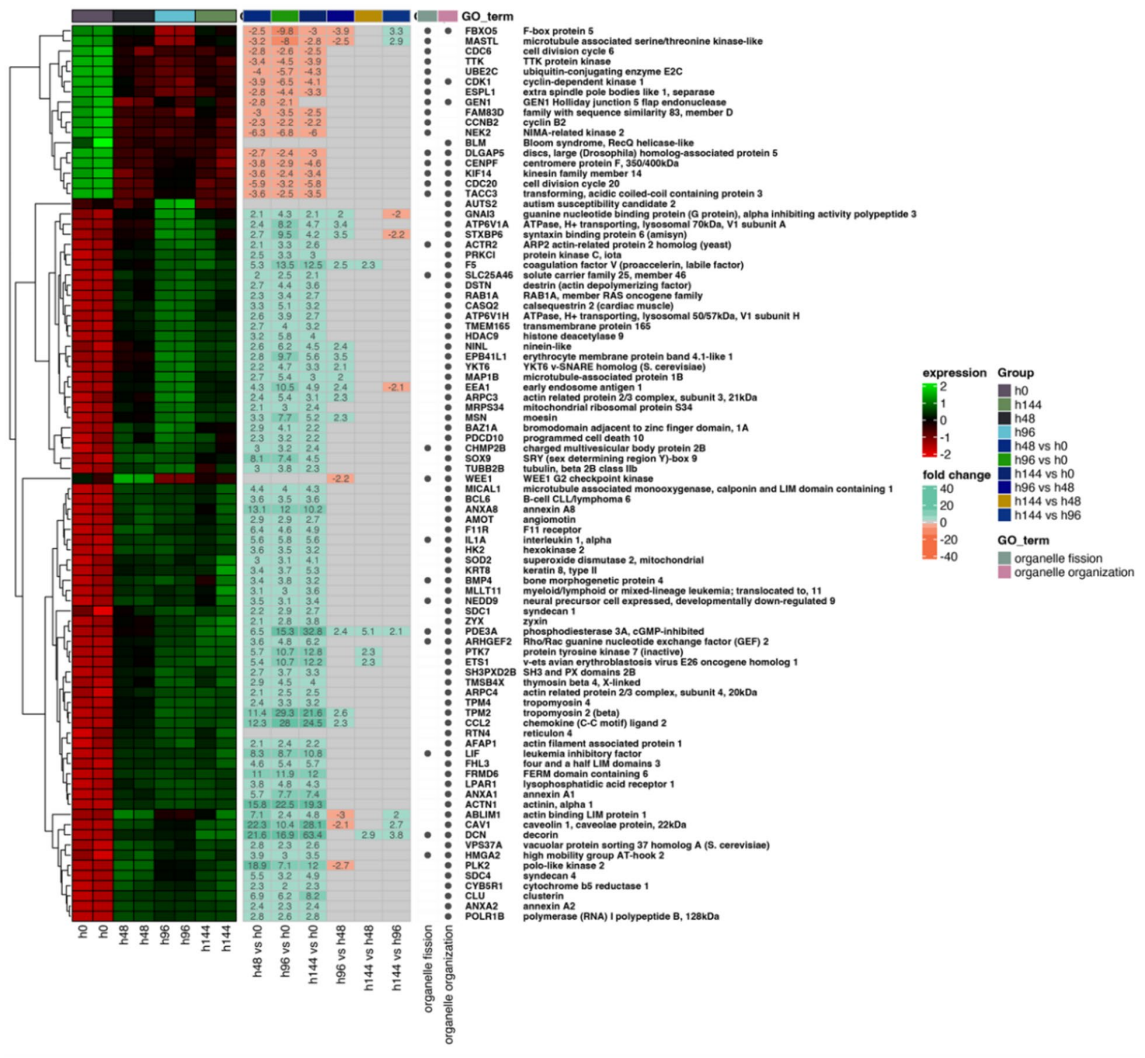
Heatmap of expressed genes related to organelle fission and organization, according to the GO term. The heatmap shows the expressed genes (left side), expression fold changes (center), and GO term ontological groups with gene names (right side) of all analyzed groups. The legends on the right side illustrate the colors used for the visualization

Next, the differentially expressed genes were classified by hierarchic clustering and visualized it as a heatmaps (Fig. 2, 3 and 4). The figures show both the expression values and expression fold changes between compared groups, according to the clustered ontological groups. It has been revealed that at the cluster related to actin filament and cytoskeleton organization (Fig. 2), the most downregulated gene across all analyzed groups was NEK2, and KIF14. Nevertheless, the expression of CAV1, TPM2, ACTN1, and CCL2 has been mostly upregulated throughout the experiment. Moreover, analysis of the cell–matrix and cell-substrate adhesion processes (Fig. 3), shows that only the KIF14 was downregulated across all analyzed groups. For the most upregulated genes, we include POSTN, FN1, ITGB8, LAMB1, ACTN1, ITGA2, and COL3A4. Furthermore, the differentially expressed genes arranged in chromosome segregation and DNA replication (Fig. 4) revealed that the expression of CCNE2 was mostly inhibited, while the expression of PLK2, and CDKN1A has been activated. Furthermore, the examination of genes of organelle fission and organization clusters (Fig. 5) indicates, that CDC20, NEK2, FBXO5, and MASTL were downregulated, while expression of PDE3A, TPM2, CCL2, DCN, CAV1, CLU, PTK7, and ETS1 has been mostly upregulated across all analyzed groups compared to control.

**Fig. 6:**
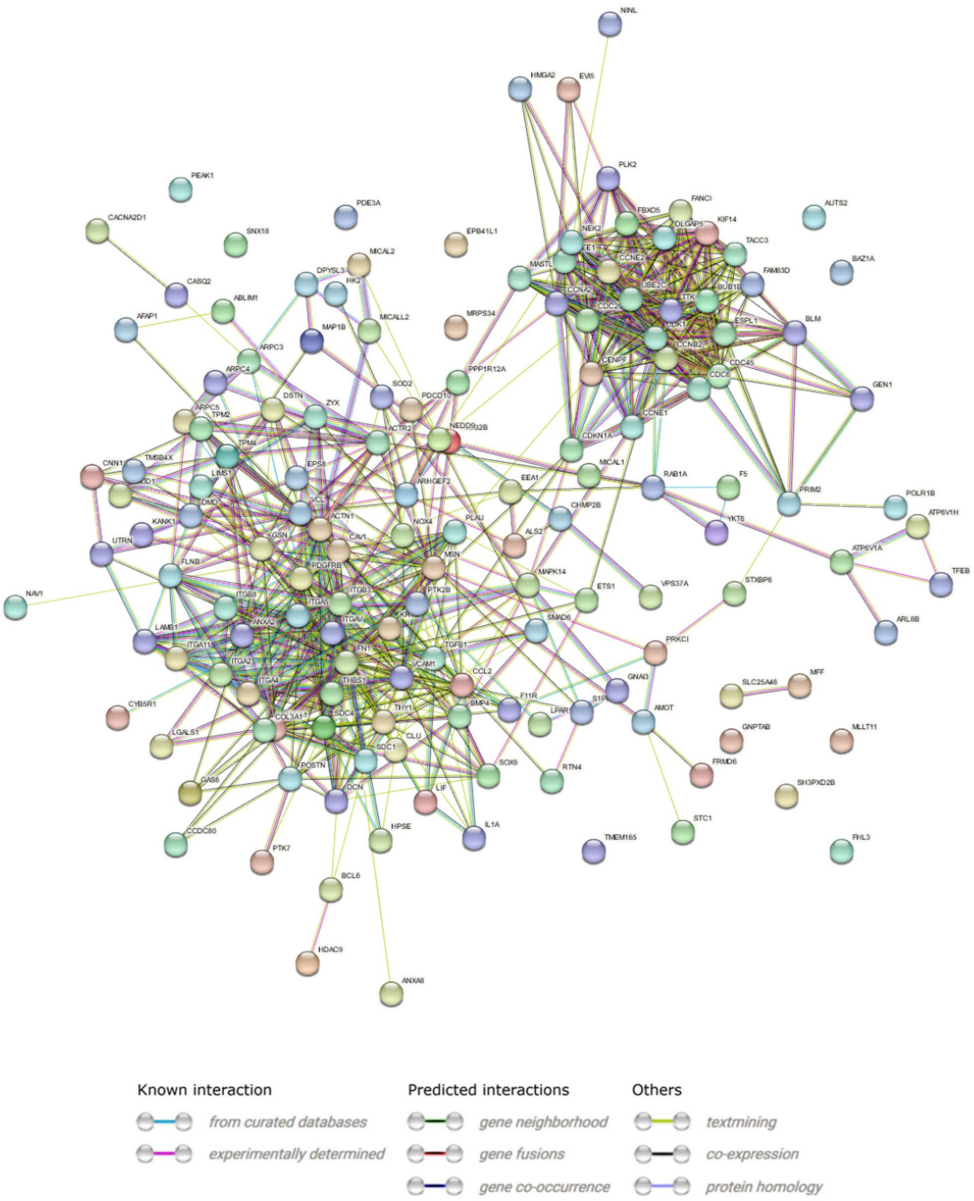
STRING-generated interaction occurrence between differently expressed genes. The intensity of the edges reflects the strength of interaction score. Proteins are shown as nodes and the color of each link defined the type of evidence available for the interaction between two proteins

Furthermore, STRING and Metascape were employed as effective online platforms to perform functional analysis of protein–protein interactions and combine it with functional enrichment interactome analysis, gene annotation, and membership search [35, 36]. For the STRING analysis, four lists containing GO BP terms were used for the differentially expressed genes (according to received heatmaps). The STRING analysis of proteins related to actin filament and cytoskeleton organization indicates that for all 148 nodes, 708 edges have been revealed (Fig. 6). The line thickness indicates the strength of data support from the sources of text mining and experiments with a cutoff value of medium confidence (0.522). The protein–protein interaction (PPI) enrichment p-value was < 10–16.

**Fig. 7:**
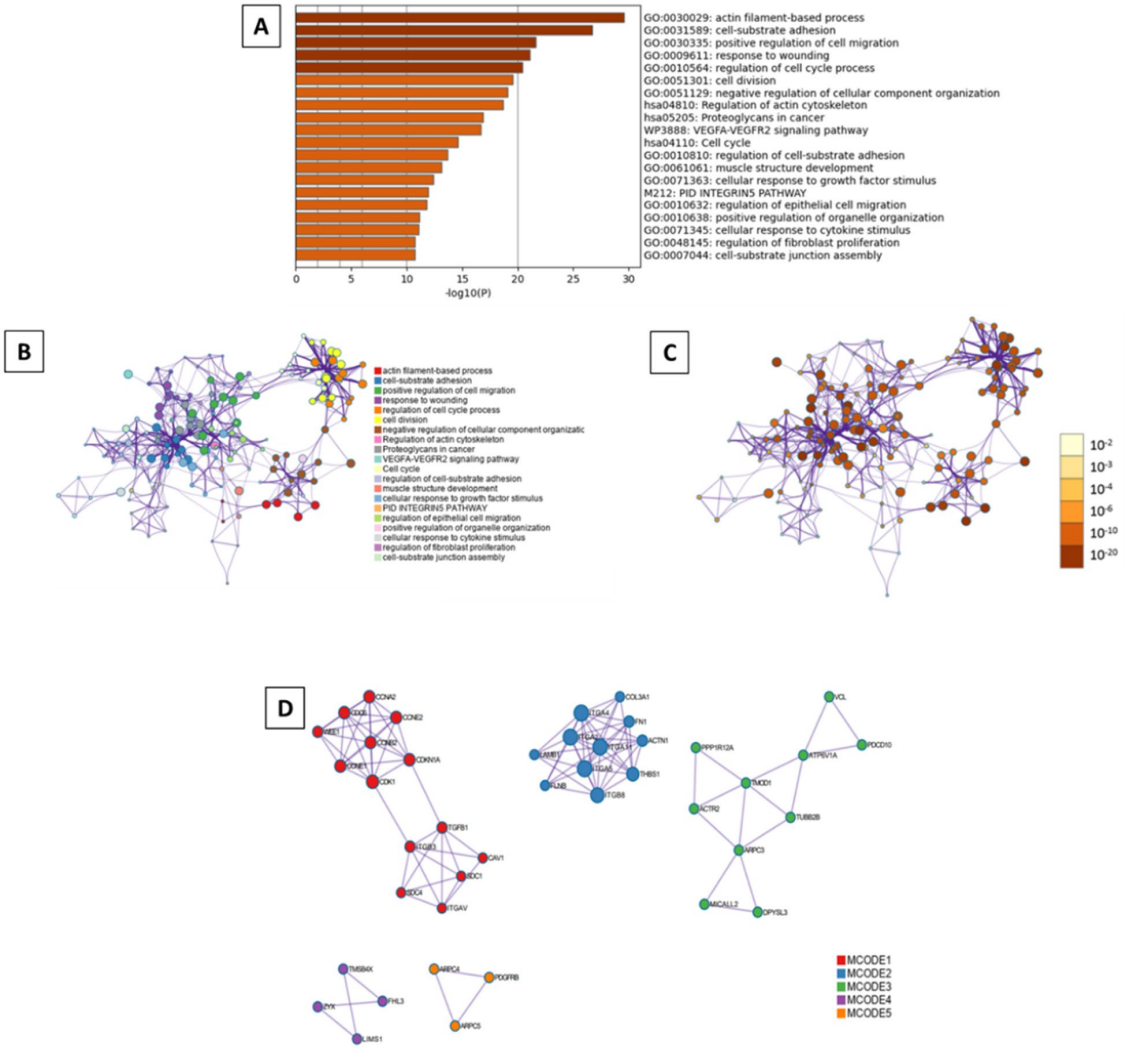
Metascape functional analysis of transcriptome profiles based on differently expressed genes. **A** Heatmap of Gene Ontology (GO) enriched terms colored by p-values. **B** Clustered network of GO enriched terms where color represent its cluster identity. A circle node represents each term, the size of node is proportional to the number of input genes fall under that term, and its color represent its cluster identity. **C** Clustered network of GO enriched terms colored by p-value, where terms containing more genes tend to have a more significant p-value. **D** Protein–protein interaction (PPI) network clustered to five most significant MCODE components form the PPI network. A circle node represents each term, the size of node is proportional to the number of input genes fall under that term, and its color represent its cluster identity

Moreover, all statistically enriched GO terms were identified, from which the most enriched were five processes: actin filament-based process (GO:0030029, log10(P) = − 29.6), cell-substrate adhesion (GO:0031589, log10(P) = − 26.7), positive regulation of cell migration (GO:0030335, log10(P) = − 21.6), response to wounding (GO:0009611, log10(P) = − 21.1), and regulation of cell cycle process (GO:0010564, log10(P) = − 20.4) (Fig. 7A). A subset of representative terms was selected from the entire cluster, converted them into a network layout (Fig. 7B, C), and applied the MCODE algorithm on this network to identify neighborhoods where proteins are densely connected (Fig. 7D).

**Fig. 8:**
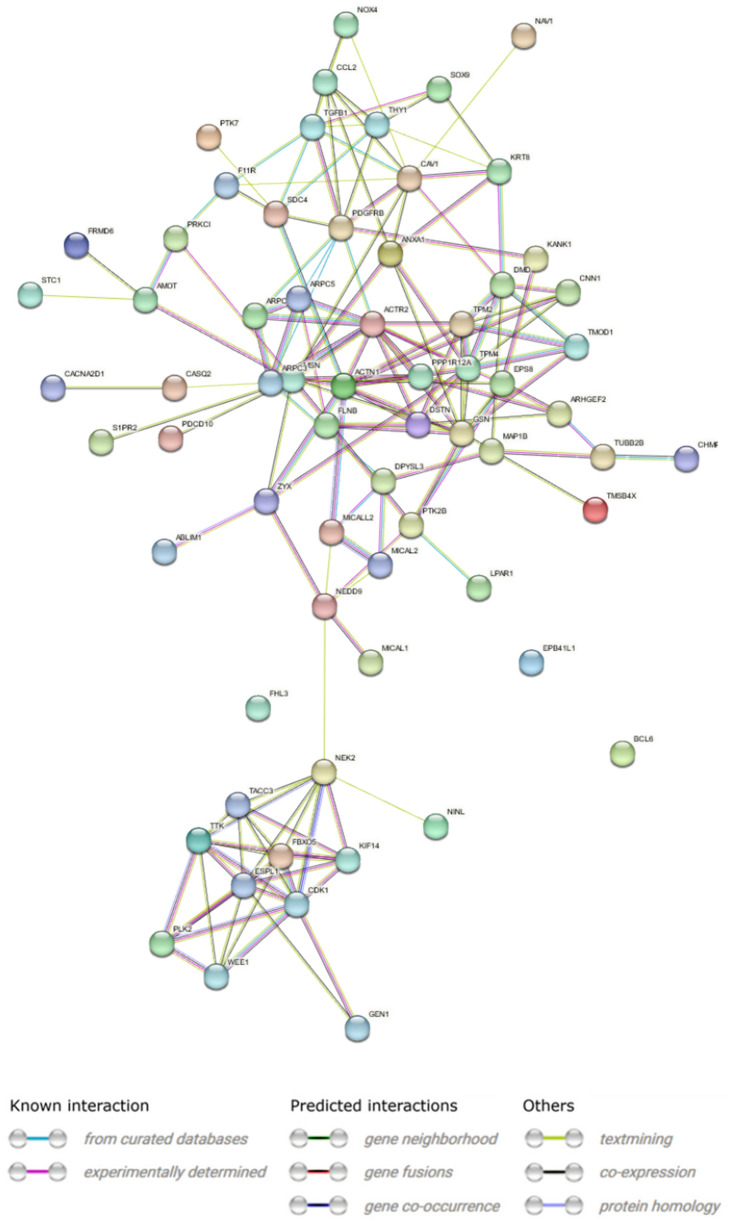
STRING-generated interaction occurrence between differently expressed genes. The intensity of the edges reflects the strength of interaction score. Proteins are shown as nodes and the color of each link defined the type of evidence available for the interaction between two proteins

**Fig. 9:**
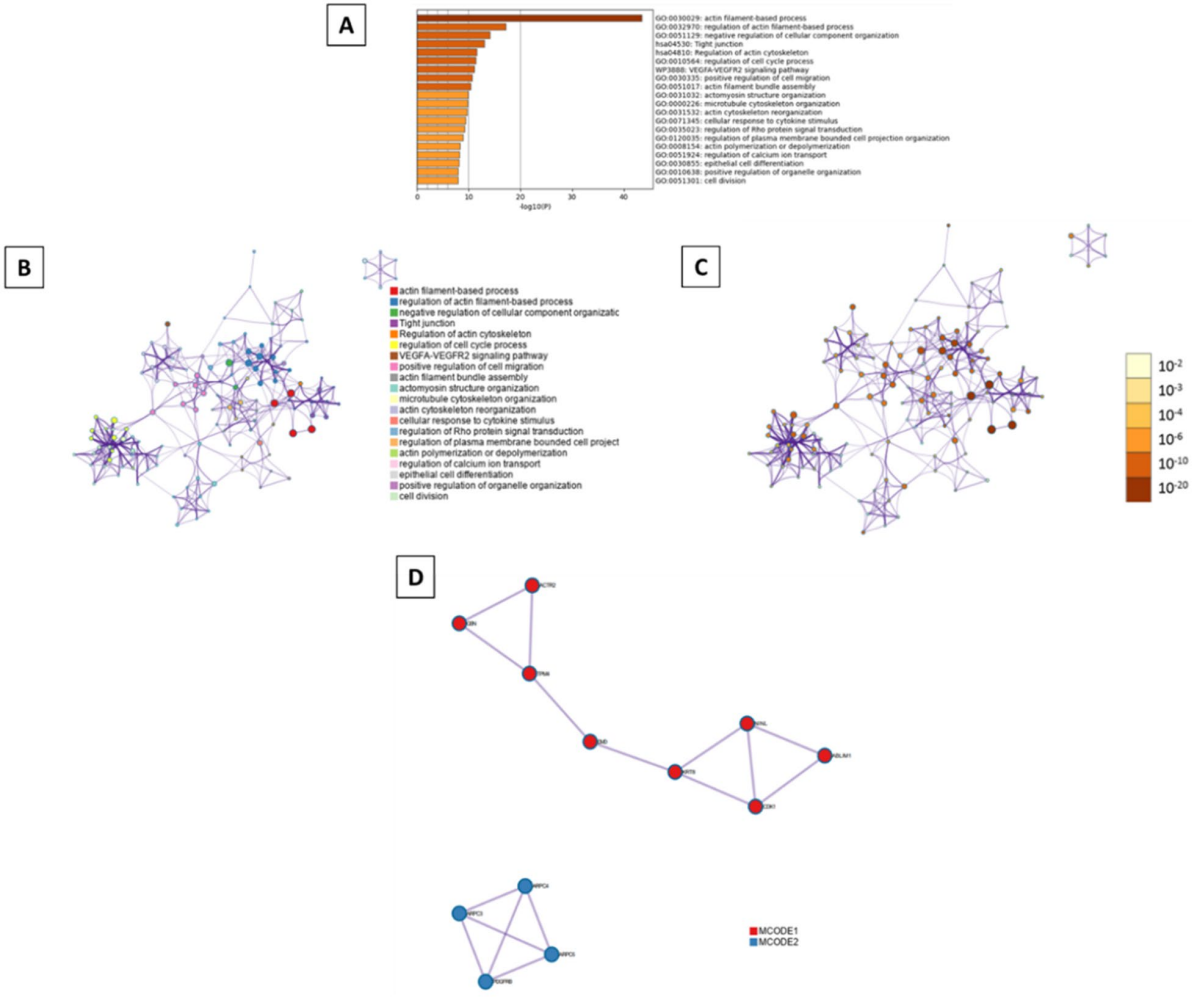
Metascape functional analysis of transcriptome profiles based on differently expressed genes. **A** Heatmap of Gene Ontology (GO) enriched terms colored by p-values. **B** Clustered network of GO enriched terms where color represent its cluster identity. A circle node represents each term, the size of node is proportional to the number of input genes fall under that term, and its color represent its cluster identity. **C** Clustered network of GO enriched terms colored by p-value, where terms containing more genes tend to have a more significant p-value. **D** Protein–protein interaction (PPI) network clustered to five most significant MCODE components form the PPI network

The protein–protein interaction of molecules related to cell–matrix and cell-substrate adhesion, according to the GO BP terms, revealed 66 nodes with a number of edges ranged 155 (Fig. 8). The medium confidence (0.55) cutoff value and the PPI enrichment p-value were < 10–16. The 20 GO terms from which the actin filament-based process (GO:0030029, log10(P) = − 43.5) was the most significant (Fig. 9A) were identified. The network layout and MCODE algorithm on this network let us identify two neighborhoods that are densely connected among analyzed proteins (Fig. 9B–D).

**Fig. 10:**
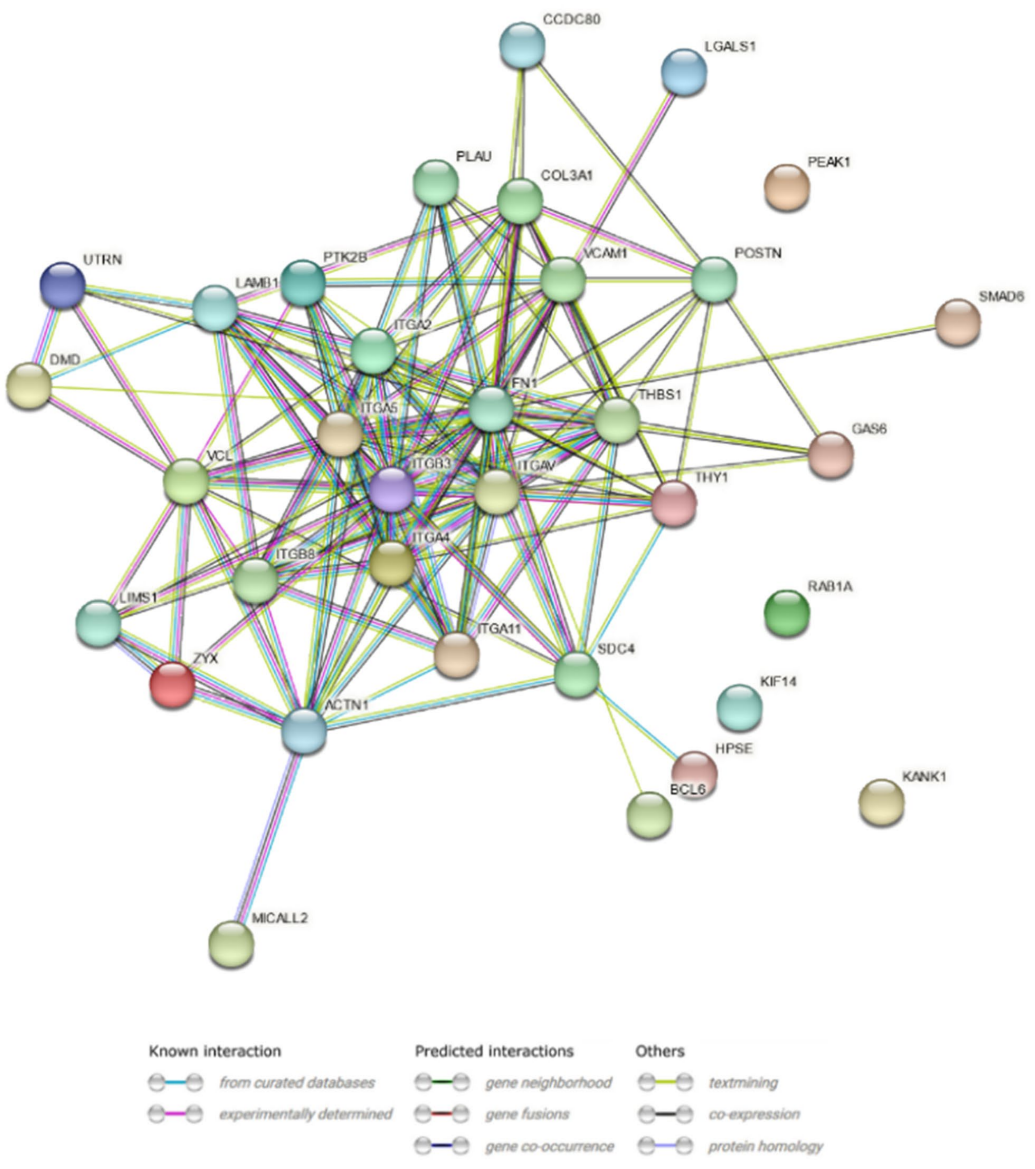
STRING-generated interaction occurrence between differently expressed genes. The intensity of the edges reflects the strength of interaction score. Proteins are shown as nodes and the color of each link defined the type of evidence available for the interaction between two proteins

The analysis of protein–protein interactions related to chromosome segregation and DNA replication performed by STRING analysis disclosed 34 nodes with 138 edges, with a cutoff value of medium confidence (0.698), and the PPI enrichment p-value was < 10–16 (Fig. 10).

**Fig. 11:**
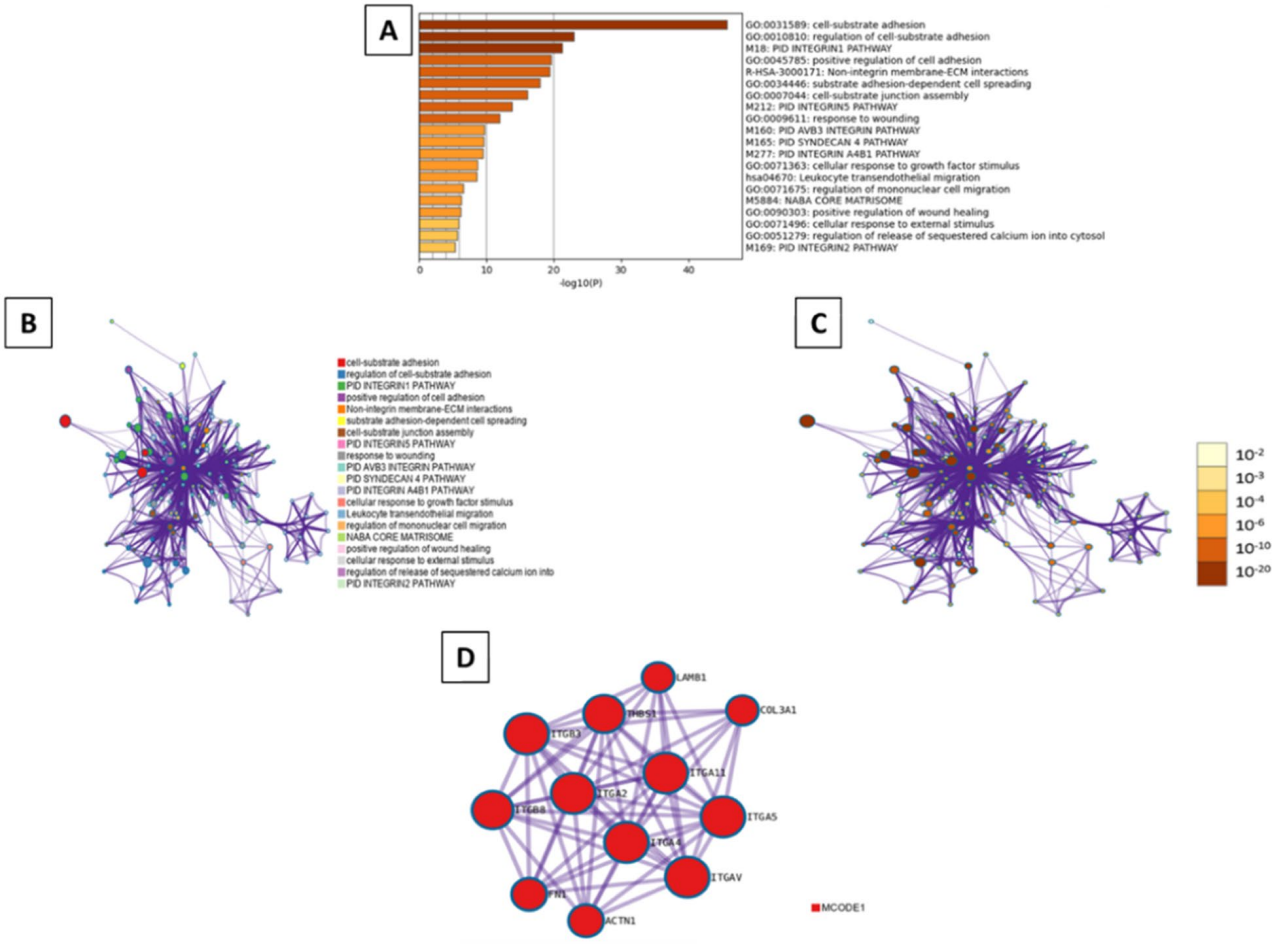
Metascape functional analysis of transcriptome profiles based on differently expressed genes. **A** Heatmap of Gene Ontology (GO) enriched terms colored by p-values. **B** Clustered network of GO enriched terms where color represent its cluster identity. A circle node represents each term, the size of node is proportional to the number of input genes fall under that term, and its color represent its cluster identity. **C** Clustered network of GO enriched terms colored by p-value, where terms containing more genes tend to have a more significant p-value. **D** Protein–protein interaction (PPI) network clustered to five most significant MCODE components form the PPI network

The Metascape analysis indicates that the most enriched GO processes were cell-substrate adhesion (GO:0031589, log10(P) = − 45.7), regulation of cell-substrate adhesion (GO:0010810, log10(P) = − 23), and PID integrin1 pathway (M18, log10(P) = − 21.2) (Fig. 11A). Moreover, the selected subset of representative terms from the full cluster and converted them into a network layout revealed 20 different biological processes, according to the node colors, while the MCODE algorithm defined only one neighborhood (Fig. 11B–D).

**Fig. 12:**
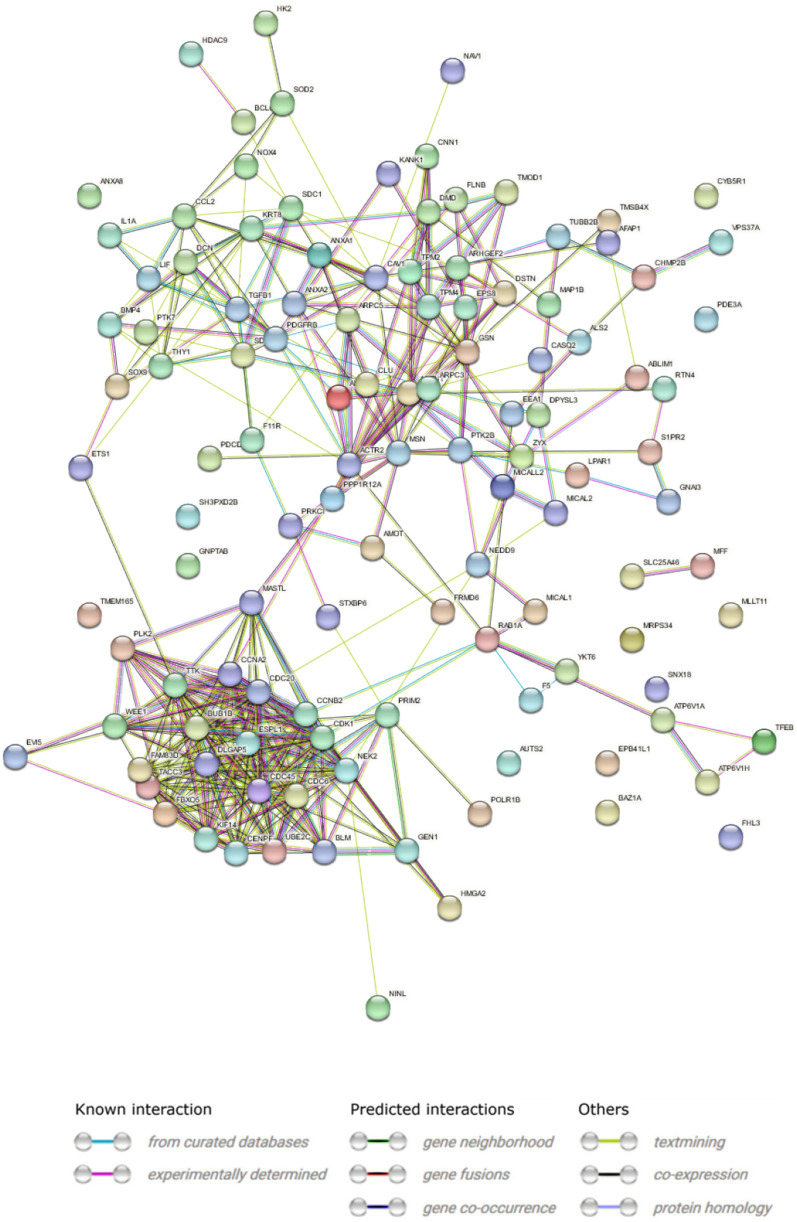
STRING-generated interaction occurrence between differently expressed genes. The intensity of the edges reflects the strength of interaction score. Proteins are shown as nodes and the color of each link defined the type of evidence available for the interaction between two proteins

The analysis of protein interactions belonging to organelle fission and organization indicates 117 nodes with 389 edges, with a cutoff value of medium confidence (0.485), and the PPI enrichment p-value was < 10–16 (Fig. 12).

**Fig. 13:**
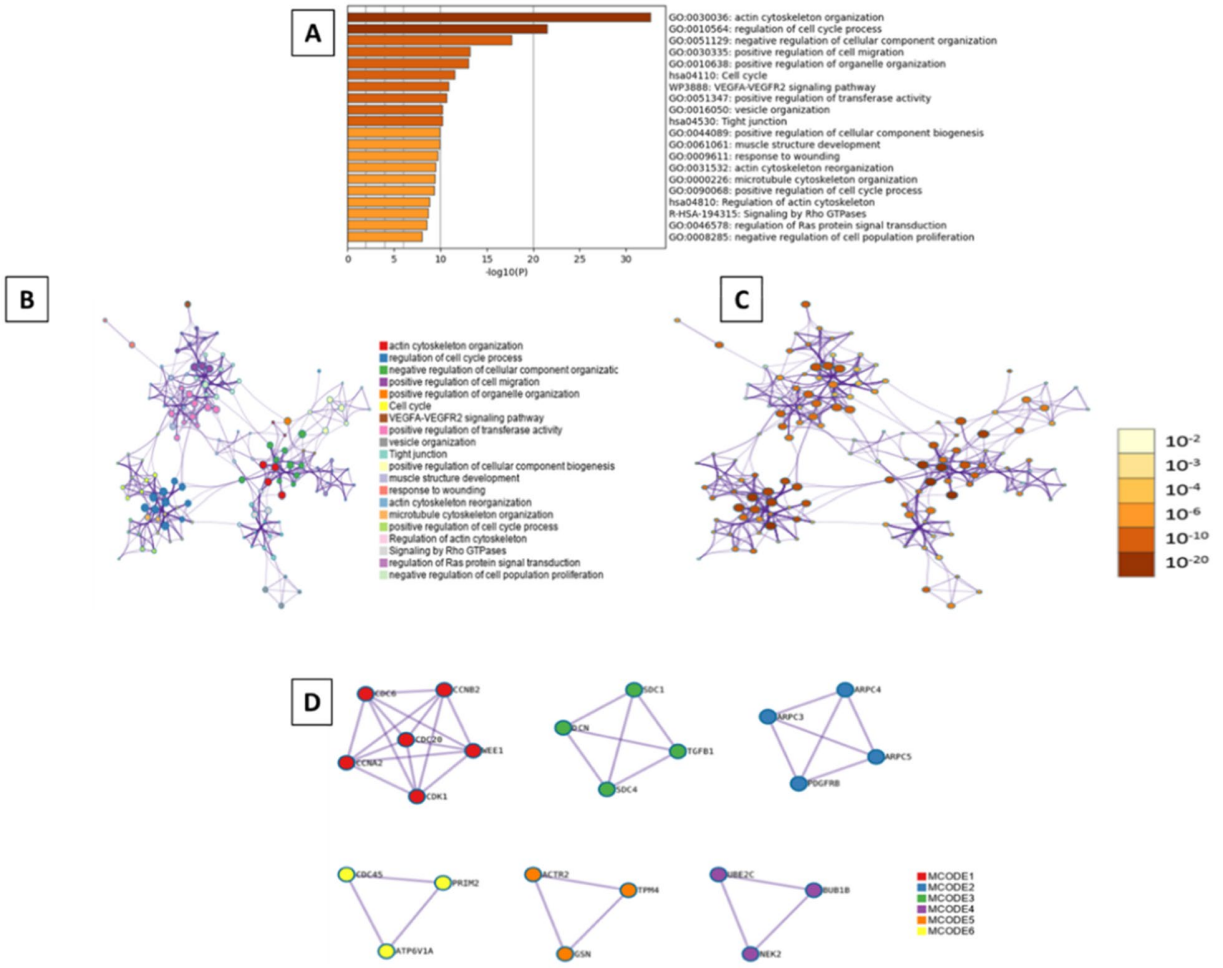
Metascape functional analysis of transcriptome profiles based on differently expressed genes. **A** Heatmap of Gene Ontology (GO) enriched terms colored by p-values. **B** Clustered network of GO enriched terms where color represent its cluster identity. A circle node represents each term, the size of node is proportional to the number of input genes fall under that term, and its color represent its cluster identity. **C** Clustered network of GO enriched terms colored by p-value, where terms containing more genes tend to have a more significant p-value. **D** Protein–protein interaction (PPI) network clustered to five most significant MCODE components form the PPI network

Next, the analysis of the function of selected genes revealed that actin cytoskeleton organization (GO:0030036, log10(P) = − 22.6), and regulation of cell cycle process (GO:0010564, log10(P) = − 21.5) (Fig. 13A). The network layout and MCODE algorithm on this network let us identify six neighborhoods that are densely connected among analyzed proteins (Fig. 13B–D).

In conclusion, among all analyzed groups, we observed commonly activated biological processes like actin filament-based process (GO:0030029) and regulation of cell cycle process (GO:0010564).

**Fig. 14:**
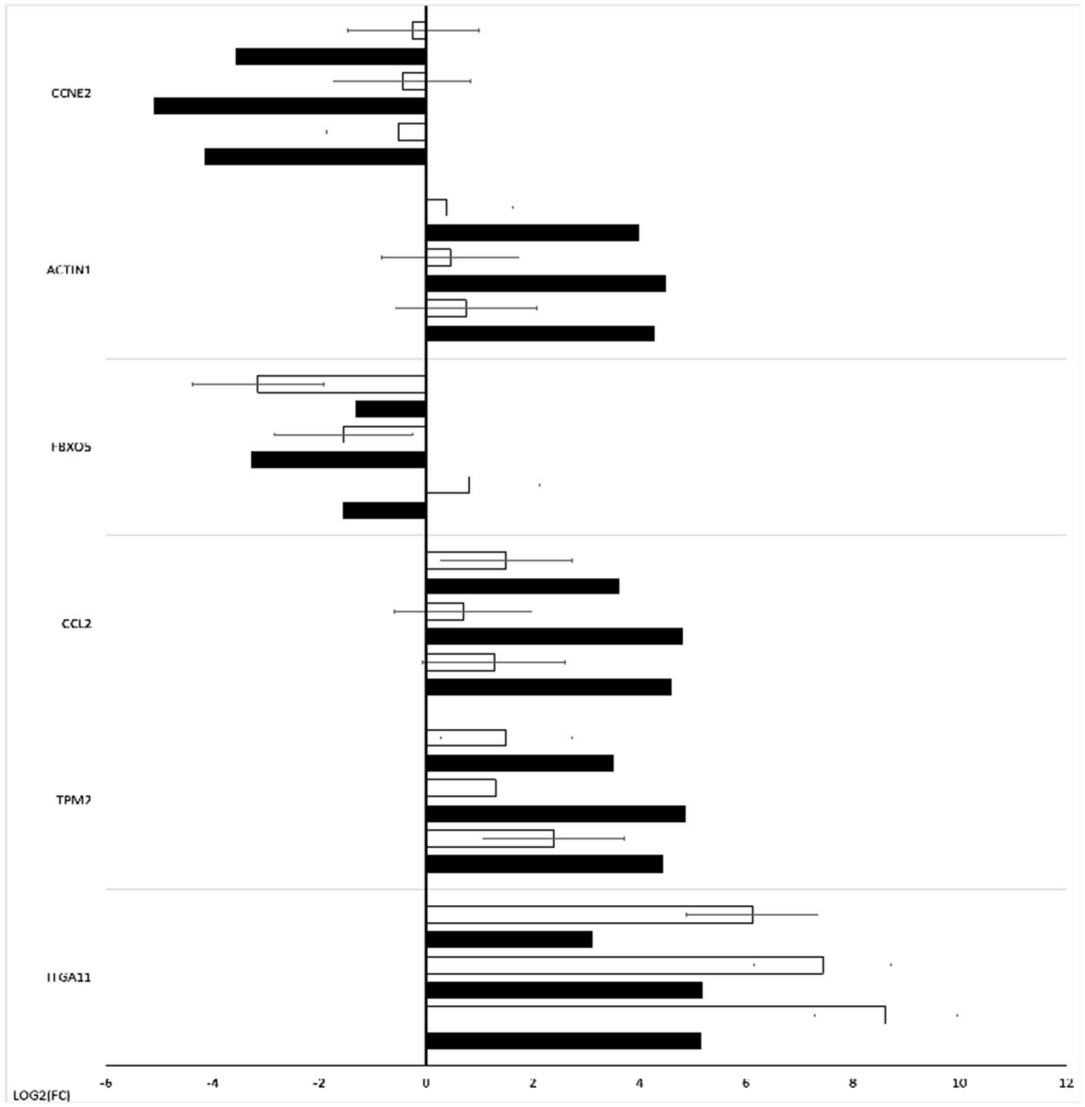
Bar graph showing the microarray validation results obtained by RT-qPCR. Black bar indicates results of microarray expressions, white bar indicates results of RT-qPCR

Results from microarray expression were confirmed by quantitative RT-qPCR. These data sets were collected, compared, and presented as a bar graph (Fig. 14). 6 selected genes were validated. The RT-qPCR result can be more representative because this method has greater quantitative precision, as opposed to the whole transcriptome analysis provided by microarrays.

## Discussion

As a dynamically changing structure, the cytoskeleton influences cellular metabolism by adjusting individual components depending on signals received from the external environment. Intercellular signaling based on chemical signals is well established, but signaling based on physical signals (including through the cytoskeleton) requires further study [1]. Knowledge of the expression of genes related to the internal environment of the cell extends the knowledge needed to understand the molecular mechanisms. Taking into account the involvement of cytoskeleton in processes related to cell differentiation and division [32, 33], the transcriptome results can be used to conduct further studies related to stemness potential of GCs [37, 38], among others from proteomic and metabolomic aspects. Demonstration of the expression of particular genes connected with cell-to-cell and cell-to-environment signaling relevant in the context of reproduction is valuable for conducting research on assisted reproduction techniques conducted in vitro. The 10 upregulated genes (ITGA11, CNN1, CCl2, TPM2, ACTN1, VCAM-1, COL3A1, GSN, FRMD6, PLK2) and 10 downregulated genes (KIF14, TACC3, ESPL1, CDC45, TTK, CDC20, CDK1, FBXO5, NEK2—NIMA, CCNE2) were selected for further evaluation.

**Fig. 15:**
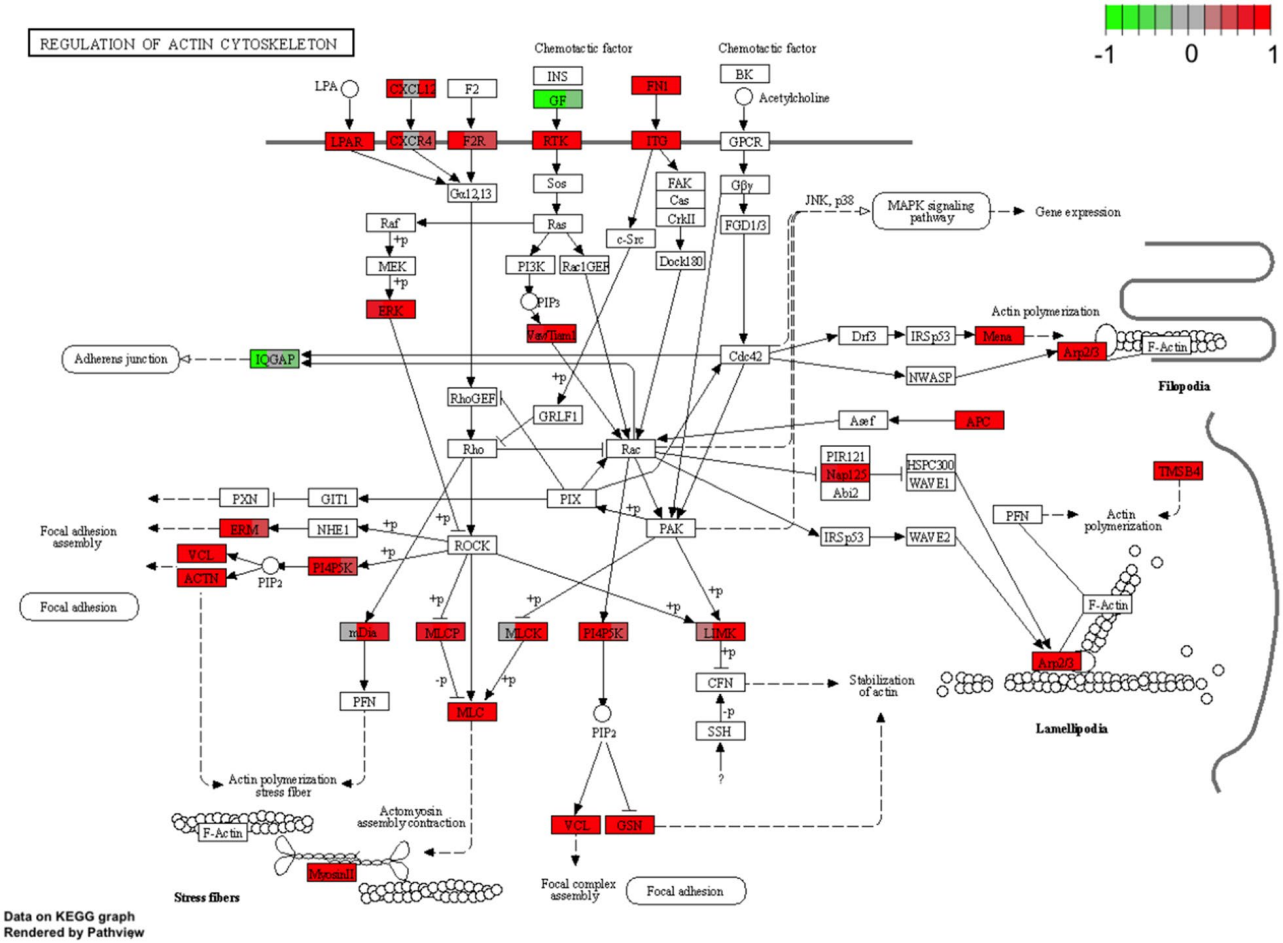
Regulation of actin cytoskeleton pathway, KEGG pathway. KEGG, Kyoto Encyclopedia of Genes and Genomes. Copyright permission obtained

The main protein building block of the cytoskeleton is actin, which can assume different conformations in the cell [39]. Spontaneous polymerization of actin molecules builds the cytoskeleton. Depending on its conformation, actin is involved in various processes within the cell, including cellular structure, motility and intracellular transport [39]. Actin-binding proteins play an important role in actin remodeling in the cytoskeleton regulating almost every aspect of actin filament formation, including maintaining a large pool of actin monomers available for polymerization, initiating the formation of new filaments, promoting elongation [39–41]. The actin-binding proteins whose expression was determined in our study are: CNN1 (calponin 1), TPM2 (tropomyosin 2, beta), ACTN (actinin, alpha 1), GSN (gelsolin). The CH (calponin homology) domain is one of the most common in animal cells, being responsible for actin cytoskeleton organization, activation of signaling pathways and calcium metabolism. The following three domains are distinguished: CH1—CH2 occurring in tandem and CH3 [42]. Point mutations present in diseases have been shown to affect the affinity of the CH1-CH2 domains for F-actin [43]. A large role has recently been attached to the role of the CH domain in cancer, involving the Rho/ROCK1 signaling pathway [44]. To date, specific expression of CNN1 in muscle tissue has been demonstrated, linking this protein to muscle contraction [45]. The role of CH domain is not limited to actin binding and cytoskeleton remodeling, it has been shown that CH domain of calponin binds to ERK (extracellular regulated kinase) leading to signal transduction between cytoskeleton and extracellular matrix [42]. CNN1 gene expression was first demonstrated in porcine granulosa cells in the present study. Highlighting the importance of calponin, it has been shown to bind to many proteins found in the cytoskeleton, including tropomyosin, actinin, and gelsolin [45]. The cytoskeleton protein TPM2 binding to actin filaments protects them from degenerative action of cofilin, maintaining a stable cytoskeleton structure [39]. Elevated expression of this gene was previously shown in the granulosa of pre-ovulatory follicles in mice [46]. Additionally, tropomyosin beta has been shown to be down-regulated in granulosa cells of women with PCOS (Polycystic ovary syndrome) [47]. Actinin (ACTN1) belongs to actin filament cross-linking proteins [2]. This protein indirectly affects cytokinesis, cell adhesion and migration without affecting actin assembly. Actinins affect cell–matrix signaling through activation of PI3K and also cell-to-cell signaling through integrins and intercellular adhesion molecules (ICAM) [48]. To date, expression of ACTN1 and TPM2 genes has been demonstrated in human granulosa cells [49]. A cytoskeletal protein showing a major influence on the composition of actin filaments is gelsolin (GSN) [39]. Gelsolin is composed of two to six domains, and the activity of breaking the connections between actin molecules is dependent on calcium concentration [50]. Mutations in this gene have been shown to cause multiple diseases [51]. It has been described that gelsolin is also associated with ovarian disease in humans [52–54], and GSN has been identified as a marker for these disease processes [53]. It has been described that increased GSN gene expression is associated with suppression of apoptotic processes, whereas downregulation is associated with increased apoptosis and this protein is also recognized as a therapeutic target [50]. Differentiation of this gene expression in mice between mural granulosa cells and cumulus cells was also demonstrated [55]. As transmembrane proteins, integrins are responsible for transmitting signals between cells by influencing the composition of the cytoskeleton through interactions with GTPases [56]. The signaling occurs through the connection between the actin cytoskeleton and the extracellular matrix [57]. It has been shown that ITGA11 expression in rat uterine endometrium is dependent on progesterone, but also on miR-126a-3p [58, 59], which by affecting its expression may be involved in embryo implantation and pregnancy maintenance. The importance of progesterone has been confirmed in studies on pregnant sows [60] and expression of the ITGA11 gene in the porcine ovary during different phases of the sexual cycle [61]. A marked increase in ITGA11 gene expression during differentiation of murine satellite cells (MSCs) into muscle cells has been described [62], which may be important in the context of the described differentiation potential of granulosa cells. ITG, GSN, ACTN genes have been shown to be involved in regulation of actin cytoskeleton. ITG affects signaling pathways, including FAK, thus regulating the activity of Rho GTPases, including Rac. This in turn leads to stabilization of actin filaments by gelsolin (GSN). An important element regulated by Rho proteins is ROCK (Rho-kinase), which by regulating ACTN transcription affects actin filament polymerization. The mechanism stabilizes the cytoskeleton while regulating intercellular signaling (Fig. 15).

**Fig. 16:**
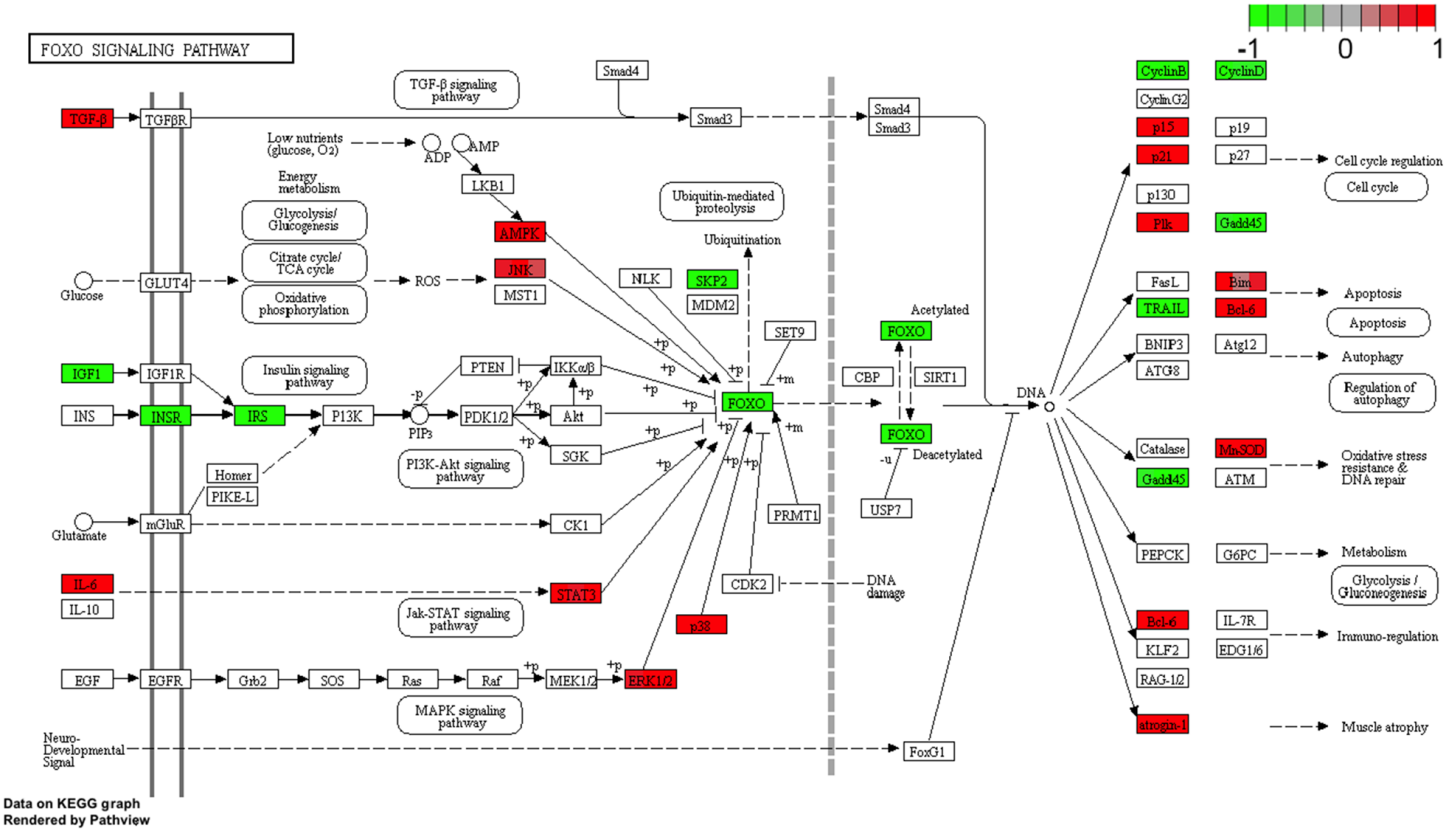
FOXO signaling pathway, KEGG pathway. KEGG, Kyoto Encyclopedia of Genes and Genomes. Copyright permission obtained

CCl2 (chemokine C–C motif ligand 2) as a chemotactic, proinflammatory substance causes the influx of white blood cells within the ovarian follicle, which is involved in the regulation of ovulation. CCl2 expression has been shown to be dependent on progesterone receptors (Pgr), which influence the expression of the Ptgs2 gene involved in PGE2 synthesis leading to a physiologically controlled inflammatory response necessary for ovulation to occur [63]. Expression of the CCl2 gene in bovine granulosa cells is also described, demonstrating its role in the gaining of competence by the oocyte [64]. In addition, it has been described that CCl2 in human granulosa shows a role in luteolysis by affecting macrophage infiltration into the corpus luteum [65]. CCl2 in combination with BMP15 affect apoptosis of porcine cumulus granulosa cells has also been described [66]. VCAM-1 is identified as a molecule involved in cell adhesion, regulates inflammation-related vascular adhesion and transendothelial migration of leukocytes such as macrophages and T lymphocytes [67] suggesting the involvement of VCAM-1 in luteolysis,. Additionally, VCAM-1 has been shown to be associated in cancer [68], autoimmune diseases [69]. VCAM-1 has been suggested to be upregulated in PCOS (Polycytic ovary syndrome) [70], where it has been shown that this gene correlates with androgen production in theca cells of mouse ovaries, [71]. Ovarian processes such as oogenesis, folliculogenesis and ovulation require close cooperation between the cytoskeleton and the extracellular matrix (ECM) which is encoded by genes described in porcine granulosa cells [72]. It is worth noting the role of COL3A1, an ECM component protein in folliculogenesis, whose expression has been demonstrated within the ovary in both cattle and pigs, mono- and polyovulatory animals [73]. COL3A1 expression in PCOS [74] as well as POI [75] suggest that Lnc-GULP1-2:1 (long non-coding RNA) could be used to alter COL3A1 expression, treating it as a therapeutic target. Additionally, COL3A1 has been shown to be a biomarker for ovarian cancers [76] and also its elevated expression reduces the effects of anticancer drugs in vitro [77]. The control of mitotic and meiotic cell division is carried out by polo-like kinases (PLKs). One of them is PLK2, which also affects cell shape and cell death [78]. It was also shown that PLK2 activity in rat granulosa cells is influenced by hormonal induction of both LH and hCG [79]. Increased expression of PLK2 shown in the above work affects cell cycle arrest in granulosa cells, which is necessary for their luteinization in the perovulatory period. The FRMD-6 protein belongs to the FERM superfamily of proteins and has been shown to be involved in the regulation of the Hippo signaling pathway [80]. This pathway has been shown to significantly affect ovarian follicle development and ovulation in cattle [81, 82]. Additionally, the Hippo signaling pathway is dependent on actin remodeling [83]. Cell proliferation in animal organisms depends on cell division, which must occur in an orderly manner and regulated by multiple mechanisms. The cell cycle is divided into individual phases associated with cell division and growth, consisting of G1, S, G2, M. Only the proper transition between the different stages of cell division allows proper karyo and cytokinesis, which is further controlled by checkpoints. Many genes have been described whose protein products are responsible for normal cell division and are also involved in controlling the succession of the different phases of oogenesis [21]. If abnormalities occur during mitosis or meiosis, this can lead to cell dysfunction, cell death and uncontrolled cell division leading to cancer [84, 85]. Many cancers within the ovary have been described that are associated with abnormal cell division within the ovary [86, 87]. In relation to this type of disorders, knowledge of gene transcription associated with cell division is valuable because it may explain the basis of many diseases and also highlight potential therapeutic targets. All of the down-regulated expression genes are cell cycle related and have been described to be involved in cell division. Interestingly, a similar direction of expression of genes responsible for cell division was obtained in in vitro experiments on human [88] and porcine granulosa cells [89]. These genes affecting cell proliferation within the ovary affect granulosa cell function [90, 91], while at the same time may cause many diseases within the ovary [86, 87]. A particularly important gene associated with cell division is CDK1, which binds to CCNs cyclins and is responsible for regulation of cell cycle events, including transition between G1, S, G2, M phases [21]. Both CDKs and CCNs have been shown to influence granulosa cell proliferation by affecting the MAPK and ERK pathways [92]. Deletion of the CDK1 gene causes early embryonic death in mice [21], and its expression in pig granulosa cells is modulated by the RSPO2 gene involved in the WNT signaling pathway [93]. Regulation of pig granulosa cell proliferation is associated with the CCNE2 gene [89] but also by numerous miRNAs [94]. Recent studies support a large role for cyclins in granulosa cell growth, which was confirmed by targeted suppressive effects of miRNAs on CCND2 [95]. There are many interactions between genes responsible for cell division such as the functional interaction between the CCNE2 and NEK2 genes [89]. Although NEK2 has previously been shown to interact with many other genes while demonstrating its involvement in cancer treatment within the ovary [96]. Continued research on treatment resistance caused by NEK2 confirms its important role in this regard and identifies this gene as a therapeutic target [97]. However, the activity of NEK2 as a kinase in granulosa cells derived from healthy porcine ovarian follicles was blocked [91]. It has been shown that the FBXO5 gene may be a prognostic biomarker in breast cancers, thus providing a potential therapeutic target [98]. In addition, the demonstrated interaction between FBXO5 (Emi1) and CDC20 confirms their role in regulating cell division through APC (anaphase promoting complex) inhibition [99]. The TGF-β/SMAD signaling pathway (Fig. 16) has a very important role in regulating ovarian function, where SMAD4 plays a large role within SMAD [100]. It was shown that silencing of SMAD4 caused an increase in many key cell cycle markers, including CDK1 and also cyclins (CNNA2, CNNB1 and CNNB2) confirming its role in granulosa cell proliferation. Additionally, SMAD4 silencing resulted in upregulation of the expression of other important cell cycle checkpoints, including CDC20 and CDC45 [100].

CDC20 has also been shown to play an important role in meiosis occurring in the oocyte, where detected mutations within this gene lead to infertility in women [101]. Interestingly, exogenous administration of gonadotropins has been shown to downregulate expression in human granulosa cells of both the CDC20 and CDC45 genes [102]. Genes closely involved in cell cycle regulation are TTK, ESPL1, TACC3, KIF14. They have been shown to be expressed in ovarian, uterine, breast, lung and colorectal cancers [84, 85, 103–106]. The available literature shows that these genes are potential therapeutic targets in cancer and determine resistance to treatment [84, 85, 103–106]. These data emphasize the necessity of understanding the expression of genes responsible for cell cycle regulation and constituting checkpoints in cells associated with the reproductive system of animals, including granulosa cells.

## Conclusions

Successful animal and human reproduction is largely dependent on interactions that occur between granulosa cells and the oocyte. Knowledge of the mechanisms and interactions occurring between the cytoskeleton and the external environment of the cells that make up the ovarian follicle provides an opportunity to understand the basis of diseases occurring within the ovary. The cytoskeleton has been shown to influence the composition of the extracellular matrix [26]. Many glycoproteins found in the ECM of granulosa cells have been described to influence the occurrence of diseases within the ovary, including PCOS and POI [107–109]. Interestingly, several genes encoding cytoskeleton-related proteins have also been described in relation to PCOS, including: TPM2, VCAM-1 [47, 70], and in the case of COL3A1 also in the aspect of POI [74, 75]. Given some ability to modulate cytoskeleton composition with actin-binding proteins [41, 110], it should be possible to influence ECM composition. Such an approach seems reasonable as a possibility to apply targeted therapy. Equally important in the context of reproductive disorders are the cell mechanisms involved in cell division, both within granulosa cells and within the oocyte [64, 90, 91]. Noteworthy are the cell division checkpoints, which, if not working properly, may impede cell replication. The TTK, ESPL1, TACC3, KIF14 genes first described in porcine granulosa cells provide new information about their cell cycle regulation. The results may provide a basis for further research on their use as a therapeutic target.

## Methods

### Animals

A total of 40 crossbred Landrace gilts with a median age of 170 days and weight of 98 kg were used in this study. All animals were housed under identical conditions. The animals in the study reached sexual maturity at 4–6 months of age and were in the follicular phase of sexual cycle.

### Collection of porcine ovarian granulosa cells

Ovaries (n = 80) were recovered at slaughter and transported to the laboratory at 38 °C in 0.9% NaCl within 30 min of harvest. In the laboratory, the ovaries of each animal were placed in PBS supplemented with fetal bovine serum (FBS; Sigma-Aldrich Co., St. Louis, MO, USA). Thereafter, single preovulatory large follicles, with a diameter estimated greater than 5 mm (n = 300), were opened into a sterile Petri dish by puncturing using a 5 ml syringe and 20 G needle, and the cumulus-oocyte complexes (COCs) and follicular fluid (FF) were recovered. The transcriptomic profile of mural GCs, which constitute a significant majority among the GCs population was analyzed. The follicular fluid was used to isolate GCs, whereas the COCs were discarded. The extracted follicular fluid after discarding COCs was filtered through sterile nylon cell strainers with a mesh diameter of 40 µm (Biologix Group, Shandong, China) to eliminate tissue debris and larger cell aggregates (including blood cells) or epithelium. The resulting suspension was centrifuged at room temperature for 10 min, 200 rpm, to obtain individual cell fractions. The GCs pellet was then resuspended in collagenase type I solution (Gibco, Thermo-Fischer Scientific, Waltham, MA, USA) 1 mg/1 mL DMEM and incubated 10 min in a 37 °C water bath and centrifuged (under the same conditions). The cell pellet was resuspended in culture medium to establish in vitro culture under the conditions described below. Granulosa cells collected from ovarian follicles were pooled to homogenize the sample.

### In vitro primary culture of porcine granulosa cells

A primary in vitro culture model was used in this study with four time intervals. For microarray expressions, cultures were maintained in two biological replicates for each time interval. For validation by RT-qPCR, cultures were maintained in a triplicate biological sample model for each time interval. Primary cultures were established from GCs in four bottles with 3 × 10^6^ cells per dish (25 cm^2^ cell culture flask, TPP, Trasadingen, Switzerland). The number of cells and their viability were assessed using the ADAM Automatic Cell Counter (NanoEnTek, Waltham, MA, USA). From the cell suspension, a 20 µL sample for number and viability analysis was stained with propium iodide and examined in a fluorescence analyzer on disposable microchips. By staining the cell nuclei, the counter is able to distinguish single cells in aggregates. Only those samples with viability above 85% were used for further studies. Cells in culture were kept until culture termination when the material was collected at 0 h, 48 h, 96 h, 144 h. The culture medium was changed every 72 h.

Culture medium consisted of Dulbecco’s Modified Eagle’s Medium (DMEM, Sigma-Aldrich, Saint Louis, MO, USA), 2% fetal calf serum (FCS) (PAA, Linz, Austria), 10 mg/mL ascorbic acid (Sigma-Aldrich, Saint Louis, MO, USA), 0.05 μM dexamethasone (Sigma-Aldrich, Saint Louis, MO, USA), 200 mM l-glutamine (Invitrogen, Carlsbad, CA, USA), 10 mg/mL gentamycin (Invitrogen, Carlsbad, CA, USA), 10,000 units/mL penicillin and 10,000 μg/mL streptomycin (Invitrogen, Carlsbad, CA, USA). Cells were cultivated at 38.5 °C under aerobic conditions (5% CO2). Once the adherent cells were more than 80% confluent, they were detached with 0.05% trypsin–EDTA (Invitrogen, Carlsbad, CA, USA) for 3 min. and then passaged. When the cells were seeded into culture bottles, the shape of the cells was close to spherical, where the cells formed a suspension in the medium. After 24 h of culture, the cells became adherent to the medium, and after 48 h, the cells assumed a star-like shape. At subsequent time intervals, the GCs became wider, more fibroblast-like. The strong adherence to the dish surface, shape change, and flattening of the cells is related to the secretion of extracellular matrix components, which correlates with the increased expression of ECM-related genes during the study.

### Microarray expression analysis and statistics

The Affymetrix procedure was previously described by Trejter et al. [111] and used in studies involving porcine oviduct epithelial cells (OECs) [112–114] as well as oocytes [115–117]. Briefly cDNA was subjected from Total RNA (100 ng) (Ambion® WT Expression Kit). Obtained cDNA was biotin labeled and fragmentated by Affymetrix GeneChip® WT Terminal Labeling and Hybridization (Affymetrix). Biotin-labeled fragments of cDNA (5.5 μg) were hybridized to Affymetrix® Porcine Gene 1.1 ST Array Strip (45 °C/20 h). Then, microarrays were washed and stained according to the technical protocol using Affymetrix GeneAtlas Fluidics Station. Subsequently the array strips were scanned by Imaging Station of GeneAtlas System. The preliminary analysis of the scanned chips was performed using Affymetrix GeneAtlasTM Operating Software. The quality of gene expression data was checked according to quality control criteria provided by the software. Obtained CEL files were imported into downstream data analysis software. All of presented analyses and graphs were performed by Bioconductor and R programming language (v4.1.2; R Core Team 2021). Each CEL file was merged with a description file. A Robust Multiarray Averaging (RMA) algorithm was used to correct background.

To show the total number of up- and down-regulated genes, the principal component analysis (PCA) of filtered data set was performed and visualized using "factoextra" library [118]. Differentially expressed genes (DEGs) from each comparison were visualized hierarchic clustering of differentially expressed genes as a heatmap using "ComplexHeatmap" library [119]. The established cut-off criteria for DEGs were based on the differences in the absolute value from the expression fold change greater than 2. Functional protein partners among all input gene list were identifies using the Search Tool for the Retrieval of Interacting Genes (STRING) (version 11.5) analysis web portal (https://string-db.org/) and by Metascape [35, 36]. The score of minimum required interaction was medium confidence (0.4). While the PPI network contains more than three nodes, the Detection (MCODE) algorithm has been used to revealed clusters directly related to genes within PPI [120]. Next, according to the p-value in the generated network, MCODE created and assigned a unique colour.

### Real-time quantitative polymerase chain reaction (RT-qPCR) analysis

Total RNA was isolated from GCs in 0 h and after 48 h, 96 and 144 h in vitro culture using an RNeasy mini column from Qiagen GmbH (Hilden, Germany). The RNA samples were resuspended in 20 µl of RNase-free water and stored in liquid nitrogen. RNA samples were treated with DNase I and reverse-transcribed (RT) into cDNA. RT-qPCR was conducted in a LightCycler real-time PCR detection system (Roche Diagnostics GmbH, Mannheim, Germany) using SYBR® Green I as a detection dye, and target cDNA was quantified using the relative quantification method. The relative abundance of analyzed transcripts in each sample was standardized to the internal standard glyceraldehyde-3-phosphate dehydrogenase (GAPDH). For amplification, 2 µl of cDNA solution was added to 18 µl of QuantiTect® SYBR® Green PCR (Master Mix Qiagen GmbH, Hilden, Germany) and primers (Table 1). One RNA sample of each preparation was processed without the RT-reaction to provide a negative control for subsequent PCR.

**Table 1:** Oligonucleotide sequences of primers used for RT-qPCR analysis

Gene	Primer sequence (5ʹ-3ʹ)		Product size (bp)
CCNE2	F	GATGGTGCTTGCAGTGAAGA	216
	R	CGATGGCTAGAATGCACAGA	
FBX05	F	AAGCCTCAAAGCCTGCATTC	221
	R	TCACCTTCGAAGCACAGTCT	
ITGA11	F	GAGGCTCCACAGGAAAGTCT	151
	R	CTTCTCATCGCTGTCACTGC	
CCl2	F	TCTCCAGTCACCTGCTGCTA	185
	R	TCCAGGTGGCTTATGGAGTC	
TPM2	F	AGTTTCCCCAAGTCTCTGCA	184
	R	TCCGTCCCTTTCAGCTTCTT	
ACTIN1	F	GGCAAGATGAGAGTGCACAA	172
	R	AGATGTCCTGGATGGCAAAG	

To quantify the specific genes expressed in the GCs, the expression levels of specific mRNAs in each sample were calculated relative to PBGD and ACTB. To ensure the integrity of these results, the additional housekeeping gene, 18S, was used as an internal standard to demonstrate that PBGD and ACTB mRNAs were not differentially regulated in GC groups. The gene for 18S rRNA expression has been identified as an appropriate housekeeping gene for use in quantitative PCR studies. Expression of PBGD, ACTB, and 18S mRNA was measured in cDNA samples from isolated GCs. The statistical significance of the analyzed genes was performed using moderated t-statistics from the empirical Bayes method. The obtained p-value was corrected for multiple comparisons using the Benjamini and Hochberg’s false discovery rate.

## Data Availability

Not applicable.
